# Genetic Diversity and Incidence of Virulence-Associated Genes of *Arcobacter butzleri* and *Arcobacter cryaerophilus* Isolates from Pork, Beef, and Chicken Meat in Poland

**DOI:** 10.1155/2015/956507

**Published:** 2015-10-11

**Authors:** Iwona Zacharow, Jarosław Bystroń, Ewa Wałecka-Zacharska, Magdalena Podkowik, Jacek Bania

**Affiliations:** ^1^Department of Epizootiology and Clinic for Birds and Exotic Animals, Wroclaw University of Environmental and Life Sciences, 50-366 Wroclaw, Poland; ^2^Department of Food Hygiene and Consumer Health Protection, Wroclaw University of Environmental and Life Sciences, 50-375 Wroclaw, Poland

## Abstract

Incidence of 9 virulence-associated genes and genetic diversity was determined in 79* A. butzleri* and 6* A. cryaerophilus* isolates from pork, beef, and chicken meat. All* A. butzleri* isolates harboured the* tlyA* gene, and most of them carried* ciaB*,* mviN*,* pldA*,* cadF*, and* cj1349* genes.* ciaB* was found to occur with higher frequency in poultry if compared with pork (*p* = 0.0007), while* irgA* was more frequent in poultry than in beef (*p* = 0.007). All 6* A. cryaerophilus* isolates harboured the* ciaB* gene, while* mviN* and* tlyA* were detected in 3 out of these isolates. Only one isolate carried the* cadF* gene. All beef-derived* A. cryaerophilus* isolates carried* ciaB*,* mviN*, and* tlyA* genes.* A. cryaerophilus* isolates from chicken meat harboured* ciaB* gene only. The pork-derived isolate harboured* ciaB* and* cadF* genes. Seventy-four genotypes were distinguished within 79* A. butzleri* isolates. Nineteen from 21 isolates derived from beef and pork were found to be closely related to* A. butzleri* from chicken meat. Each of the 6* A. cryaerophilus* isolates was found to have unique genotype. We demonstrated that closely related genotypes can spread within pork, beef, and chicken meat populations of* A. butzleri* but not* A. cryaerophilus*.

## 1. Introduction

Genus* Arcobacter,* formerly known as “aerotolerant* Campylobacter*,” was included into the family Campylobacteraceae in 1991 [1] and currently consists of twenty species [2, 3]. Three* Arcobacter* species, that is,* A. butzleri*,* A. cryaerophilus*, and* A. skirrowii*, have been mainly associated with enteritis in humans and abortion in pigs [4–6].* A. butzleri* has been classified as a serious hazard to human health by the International Commission on Microbiological Specifications for Foods in 2002 [7]. The bacteria have been isolated from food, mainly from products of animal origin, with the highest prevalence in poultry, followed by pork and beef [8–10]. Consumption of* Arcobacter*-contaminated food is regarded as the most likely source of human infection [11].* Arcobacter* spp. were also isolated from drinking water reservoirs and sewage [12]. Recent evidence suggests that* Arcobacter* spp., especially* A. butzleri* and* A. cryaerophilus*, may be involved in human enteric diseases [4, 13, 14]. Infections involving* Arcobacter* spp. may result in abdominal pain with acute diarrhoea or prolonged watery diarrhoea [15].

Analysis of complete genome of* A. butzleri* ATCC (American Type Culture Collection) 49616 revealed some homologues to virulence genes identified in* Campylobacter* and other bacteria, including* E. coli* and* Vibrio* spp. Already identified virulence-associated genes of* A. butzleri* include* cadF, cj1349, ciaB, mviN, pldA, tlyA*,* irgA*,* hecA*, and* hecB* [16].* cadF* and* cj1349* encode fibronectin-binding proteins;* ciaB* encodes* Campylobacter* invasive antigen (CiaB), which may contribute to host cell invasion;* hecA* encodes HecA protein, a member of the filamentous hemagglutinin;* hecB* encodes a hemolysin activation protein;* tlyA* encodes hemolysin;* mviN* encodes essential protein MViN required for peptidoglycan biosynthesis;* pldA* encodes outer membrane phospholipase PldA associated with lysis of erythrocytes; and* irgA* encodes iron-regulated outer membrane protein IrgA. Although some authors reported on abundance of virulence factors, pathogenicity, and genetic diversity of food-derived* Arcobacter* isolates throughout the world, characteristics of* Arcobacter* spp. in Poland are still unknown [17–21].

The aim of this work was to determine the incidence of virulence-associated genes and genetic diversity of* A. butzleri* and* A. cryaerophilus* isolates from pork, beef, and chicken meat in Poland.

## 2. Materials and Methods

### 2.1. Bacterial Strains

Seventy-nine* A. butzleri* and 6* A. cryaerophilus* cultures were isolated from meat samples, purchased from retail market in Lower Silesia region (Poland) as described previously [22]. Among 79* A. butzleri* isolates, 58, 11, and 10 were obtained from chicken, beef, and pork samples, respectively. Within* A. cryaerophilus* cultures, 3 isolates were from beef, 2 were from chicken, and one was from pork meat.* Arcobacter* spp. isolates were cultured according to Houf et al. [23] with modifications, in* Arcobacter* broth (Oxoid) with selective supplement containing cefoperazone, amphotericin B, and teicoplanin (CAT, Oxoid). Additionally, novobiocin (32 mg/L), 5-fluorouracil (100 mg/L), and trimethoprim (64 mg/L) (Sigma) were added to the broth. After 48-hour incubation in aerobic atmosphere, at 30°C, the bacteria were subcultured on* Arcobacter* agar plates (supplemented with chemotherapeutics mentioned above) and in parallel on agar plates with defibrinated sheep blood (Oxoid). Phenotypically suspected colonies were transferred to blood agar plates and incubated in aerobic conditions for 48 h at 30°C. One* Arcobacter* spp. isolate per sample was taken for further characterization. The isolates were preserved by freezing in Cryobank (Mast Diagnostics) at −80°C.

### 2.2. Preparation of Bacterial DNA

Total DNA was isolated as described by Agersborg et al. [24]. Briefly, the bacteria from 1 mL overnight culture were pelleted by centrifugation and suspended in 200 *μ*L of distilled water containing 1% Triton X-100. The mixture was boiled for 10 min, and then the tubes were centrifuged for 5 min at 13 000 ×g. The supernatant containing DNA was used in PCR.

### 2.3. Species Identification of Arcobacter Isolates

The isolates were identified at species level using multiplex PCR according to Houf et al. [25]. Amplification products were resolved in 1.5% agarose containing 0.5 *μ*g/mL ethidium bromide and documented using GelDocXR System (BioRad, Hercules, CA). Each PCR run was performed using DNA from the reference CCM (Czech Collection of Microorganisms) 4826* A. butzleri* and CCM 3933* A. cryaerophilus* strains as positive controls and ATCC 33560* Campylobacter jejuni* as a negative control.

### 2.4. Identification of Virulence-Associated Genes

*A. butzleri* and* A. cryaerophilus* isolates were PCR-screened for the presence of nine virulence-associated genes, such as* cadF, cj1349*,* ciaB*,* mviN*,* pldA*,* tlyA*,* irgA*,* hecA*, and* hecB*, as described by Douidah et al. [17]. The 10 *μ*L aliquots of PCR products were resolved on 1.5% agarose gel and documented with a GelDoc XR documentation system (BioRad, Hercules, USA).

### 2.5. Genotyping of* A. butzleri* and* A. cryaerophilus* Isolates

A survey of the genetic diversity of the* A. butzleri* and* A. cryaerophilus* isolates was performed using ERIC PCR primers ERIC2 5′-AAGTAAGTGACTGGGGTGAGCG-3′ and ERIC1R 5′-ATGTAAGCTCCTGGGGATTCAC-3′ as described by Houf et al. [26]. The PCR was performed in a mixture containing 20 mmol/L Tris-HCl, pH 8.4, 3 mmol/L MgCl_2_, 50 mmol/L KCl, 0.2 mmol/L of each deoxyribonucleotide, 1.25 U of Taq DNA polymerase (Thermo Scientific, Poland), 50 pmole of each primer (Genomed, Warsaw, Poland), and 2 *μ*L of DNA solution. Thirty-six PCR cycles were carried out according to the following scheme: denaturation at 94°C for 30 s, annealing at 35°C for 30 s, and elongation at 72°C for 120 s. The denaturation/annealing and annealing/elongation ramping rates were set to 5 minutes [27]. Amplicons were separated on 3% agarose gel at 170 V for 3 h. The gels were stained with ethidium bromide, visualized on a UV transilluminator, and photographed. The resulting profiles were analyzed using the software available on http://insilico.ehu.es/dice_upgma/ to generate dendrograms by UPGMA clustering, using Dice correlation.

### 2.6. Statistical Analysis

Mann-Whitney *U* test was applied to assess statistical significance of obtained data. Calculations were performed using Statistica 9.1 (StatSoft, Poland). *p* values below 0.05 were considered to be significant.

## 3. Results

### 3.1. Prevalence of Virulence-Associated Genes in* A. butzleri* and* A. cryaerophilus* Isolates

Screening of 79* A. butzleri* isolates revealed that all of them harboured the* tlyA* gene. Most of the* A. butzleri* isolates carried* ciaB* (97%),* mviN* (97%),* pldA* (92%),* cadF* (89%), and* cj1349* (66%), while* hecB* (48%),* irgA* (46%), and* hecA* (30%) genes were less frequent (Table 1).* ciaB* was found to occur with higher frequency in poultry if compared with pork (*p* = 0.0007), while* irgA* was more frequent in poultry than in beef (*p* = 0.007).

**Figure 1:**
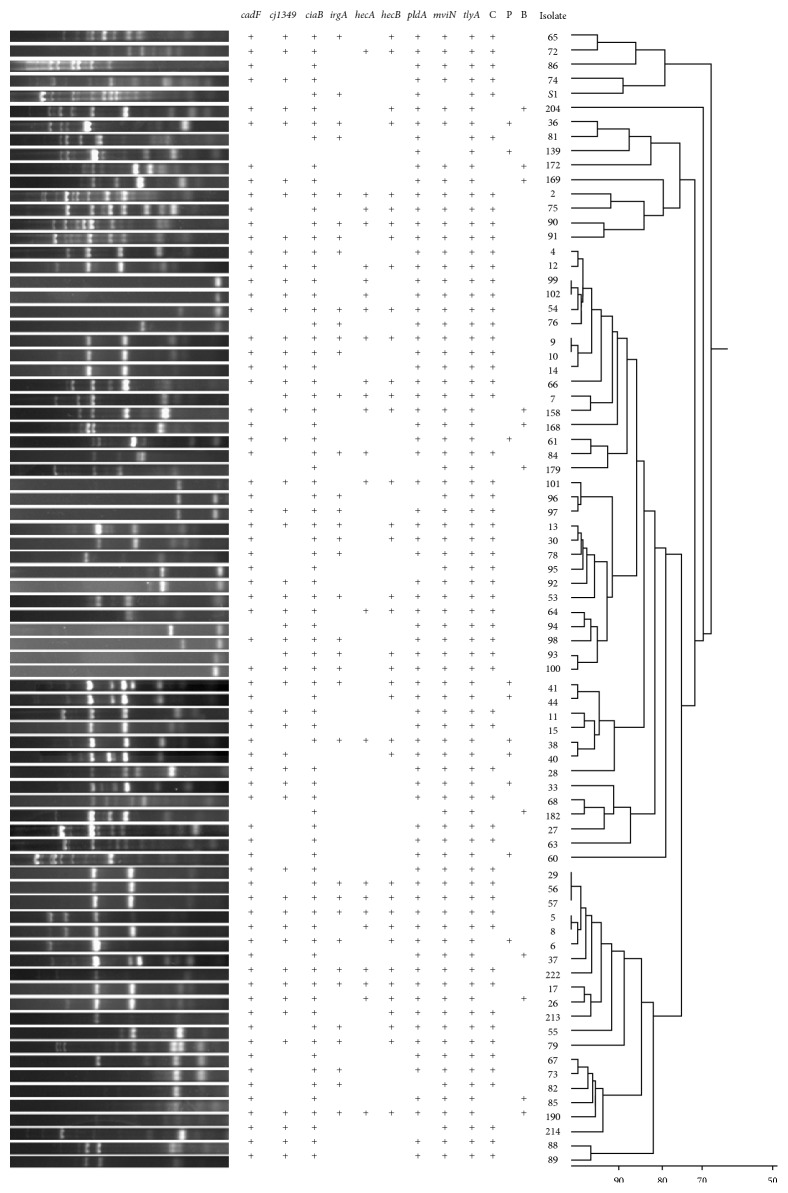
Dendrogram generated by UPGMA clustering based on PCR fingerprinting using ERIC primers performed on 79* A. butzleri* isolates. Repertoire of virulence-associated genes of each isolate was presented in left panel. Isolate origin was indicated as C, P, and B for chicken, pork, and beef meat, respectively.

Ten percent (8 out of 79) of the* A. butzleri* isolates harboured all nine genes, and most of them (7 isolates) derived from chicken meat. The carriage of 8 and 7 virulence-associated genes was reported in 44% (35 out of 79) of* A. butzleri* isolates. Most of them were found in chicken meat (28 isolates), followed by pork (4 isolates) and beef (3 isolates). Seventeen (22%), 14 (18%), and 2 (3%)* A. butzleri* isolates possessed 6, 5, and 4 virulence-associated genes, respectively (Figure 1). The isolates harbouring 3 (2 isolates) and 2 genes (1 isolate) were only reported in* A. butzleri* from beef and pork, respectively.

All* A. cryaerophilus* isolates possessed the* ciaB* gene, while* mviN* and* tlyA* were detected in 3 out of the 6 isolates. Only one isolate carried the* cadF* gene.* cj1349*,* hecA*,* hecB*,* irgA*, and* pldA* genes were not detected in this population. All the 3 beef-derived* A. cryaerophilus* isolates carried* ciaB*,* mviN*, and* tlyA* genes. Two* A. cryaerophilus* isolates from chicken meat harboured* ciaB* gene only. The pork-derived isolate harboured* ciaB* and* cadF* genes (Table 1).

### 3.2. Genotypes of* A. butzleri* and* A. cryaerophilus*

Seventy-four genotypes were distinguished within 79* A. butzleri* isolates (Figure 1). These isolates could be grouped into 8 clusters using 90% cut-off similarity. Nineteen from 21 isolates derived from beef and pork were found to be closely related to* A. butzleri* from chicken meat. Genotypes of 6 isolates from beef and 6 from pork shared >90% similarity to chicken isolates, when >80% similarity to chicken genotypes was found in 4 isolates from beef and 3 isolates from pork. Only two genotypes, one shared by beef (isolate 204) and one by pork isolate (isolate 60), were found to be distant from chicken genotypes. No relation between* A. butzleri* genotype and incidence of virulence genes could be found.

**Figure 2:**
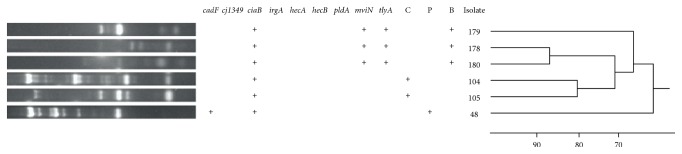
Dendrogram generated by UPGMA clustering based on PCR fingerprinting using ERIC primers performed on 6* A. cryaerophilus* isolates. Repertoire of virulence-associated genes of each isolate was presented in left panel. Isolate origin was indicated as C, P, and B for chicken, pork, and beef meat, respectively.

Each of the 6* A. cryaerophilus* isolates was found to have unique genotype (Figure 2). Over 80% genotype similarity was only found within two beef and two pork* A. cryaerophilus* isolates but not between isolates from beef, pork, and chicken meat.

## 4. Discussion

*A. butzleri* and* A. cryaerophilus* are considered as potential food-borne pathogens [28], but the knowledge on the presence of virulence-associated factors and genetic diversity of food-derived isolates is still limited. Thus, we examined the prevalence of virulence-associated genes in* A. butzleri* and* A. cryaerophilus* populations recovered from retail pork, beef, and chicken meat. The* tlyA*,* cj1349*,* mviN*,* pldA*,* cadF*, and* ciaB* genes were found to occur most frequently in* A. butzleri* isolates. Similar results were obtained by Collado et al. [18], who showed that at least 73% of* A. butzleri* isolates from shellfish in Chile harboured* cadF*,* ciaB*,* mviN*,* pldA*, and* tlyA genes*, although only 31% of the isolates possessed* cj1349* gene. In turn, in Belgium [17], Germany [29], and Iran [21], these six genes were found in all* A. butzleri* isolates recovered from various sources, including meat. A high occurrence of these genes was also reported in Spain, where all isolates from meat, mussels, and sewage possessed* ciaB* gene, when 92% of them harbored* cadF* and* cj1349* genes [30]. Consistent to other reports, we showed* hecA*,* hecB*, and* irgA* genes to occur most rarely within* A. butzleri* population [17, 18, 29, 30].

Our results indicate that 14% of* A. butzleri* isolates harboured all nine virulence-associated genes. These results are similar to that obtained by Douidah et al. [17], who found all these genes in 15% of* A. butzleri* isolates, and Karadas et al. [29], who reported 13% of* A. butzleri* isolates carrying all mentioned virulence-associated genes.

Consistent with previous reports [18, 21, 30], we noted a higher incidence of virulence-associated genes in* A. butzleri* as compared to* A. cryaerophilus*. In our study, only* ciaB* gene was detected in all the six* A. cryaerophilus* isolates,* mviN* and* tlyA* were detected in three isolates, and* cadF* gene was detected in one isolate. A high frequency of virulence genes was observed in beef-derived isolates, since all the three isolates possessed* ciaB*,* mviN*, and* tlyA* genes. A high incidence of* mviN* (100%) and* tlyA* (62%) genes was already reported in* A. cryaerophilus* derived from cattle [21]. Collado et al. [18] showed that* mviN* and* tlyA* genes commonly occur in* A. cryaerophilus* from molluscs, when Douidah et al. [17] reported* mviN* gene to dominate in* A. cryaerophilus* from human and animals. In contrast to* A. butzleri*, no* A. cryaerophilus* isolates harboured multiple virulence genes, which is consistent with results obtained by other authors [17, 18, 21]. According to Douidah et al. [17], this difference could be explained by a different pathogenic behaviour of these species or due to a high heterogeneity of their genomes.

A high diversity of virulence genes pattern was found in* A. butzleri* isolates from each source, except* ciaB* and* irgA* genes found to be more frequent in* A. butzleri* from poultry.

Relatively little is known on genetic structure of* Arcobacter* spp. population and even less on impact of genetic background on distribution of virulence-associated genes. Data obtained in this study demonstrate no relation between genotype and virulence genes repertoire in studied* Arcobacter* species. According to our data, even closely related isolates may carry different virulence genes. Similar observation was already made by Lehmann et al. [19] studying* Arcobacter* spp. genotypes from food in Germany.

Irrespective of genotypic method used, a large genetic heterogeneity of* Arcobacter* spp. population is frequently reported [20, 31–33]. However, some reports point out that closely related* Arcobacter* spp. genotypes can be isolated from some nonoverlapping sources like beef, lamb, pork, poultry, and fish meat [9, 19]. Our data also argue that genotypes closely related to* A. butzleri* from poultry meat can also be found within populations from pork and beef meat. In* A. cryaerophilus* isolates studied here, low genetic relatedness between pork, beef, and chicken meat isolates was noted, but general conclusions cannot be drawn here due to limited number of isolates.

## 5. Conclusions

Repertoire of virulence-associated genes in* A. butzleri* and* A. cryaerophilus* isolates from retail meat in Poland is similar to that reported in other countries. It seems that closely related genotypes can spread within pork, beef, and chicken meat populations of* A. butzleri* but not* A. cryaerophilus.*

## Figures and Tables

**Table 1:** Carriage of virulence genes by *A. butzleri* and *A. cryaerophilus* isolates.

Species	Origin/number of isolates	Percent of isolates harbouring virulence genes possessing specific gene								
		*cadF *	*cj1349 *	*ciaB *	*irgA *	*hecA *	*hecB *	*pldA *	*mviN *	*tlyA *
*A. butzleri *	Chicken meat/58	90	71	100	53	34	48	93	98	100
	Pork/10	90	60	80	40	10	60	100	90	100
	Beef/11	82	45	100	9	27	36	82	100	100
	Total/79	89	66	97	46	30	48	92	97	100

*A. cryaerophilus *	Chicken meat/2	0	0	100	0	0	0	0	0	0
	Pork/1	100	0	100	0	0	0	0	0	0
	Beef/3	0	0	100	0	0	0	0	100	100
	Total/6	17	0	100	0	0	0	0	50	50
